# HexAI-TJAtxt: A textual dataset to advance open scientific research in total joint arthroplasty

**DOI:** 10.1016/j.dib.2023.109738

**Published:** 2023-10-31

**Authors:** Soheyla Amirian, Husam Ghazaleh, Luke A. Carlson, Matthew Gong, Logan Finger, Johannes F. Plate, Ahmad P. Tafti

**Affiliations:** a School of Computing, University of Georgia, Athens, GA, USA; b Department of Computer Science, Quincy University, Quincy, IL, USA; c Department of Orthopaedic Surgery, School of Medicine, University of Pittsburgh, Pittsburgh, PA, USA; d Department of Health Information Management, School of Health and Rehabilitation Sciences, University of Pittsburgh, Pittsburgh, PA, USA; e Intelligent Systems Program, School of Computing and Information, University of Pittsburgh, Pittsburgh, PA, USA

**Keywords:** Total joint arthroplasty, Large scale textual dataset, Computational text analytics, ChatGPT

## Abstract

Total joint arthroplasty (TJA) is the most common and fastest inpatient surgical procedure in the elderly, nationwide. Due to the increasing number of TJA patients and advancements in healthcare, there is a growing number of scientific articles being published in a daily basis. These articles offer important insights into TJA, covering aspects like diagnosis, prevention, treatment strategies, and epidemiological factors. However, there has been limited effort to compile a large-scale text dataset from these articles and make it publicly available for open scientific research in TJA. Rapid yet, utilizing computational text analysis on these large columns of scientific literatures holds great potential for uncovering new knowledge to enhance our understanding of joint diseases and improve the quality of TJA care and clinical outcomes. This work aims to build a dataset entitled HexAI-TJAtxt, which includes more than 61,936 scientific abstracts collected from PubMed using MeSH (Medical Subject Headings) terms within “MeSH Subheading” and “MeSH Major Topic,” and Publication Date from 01/01/2000 to 12/31/2022. The current dataset is freely and publicly available at https://github.com/pitthexai/HexAI-TJAtxt, and it will be updated frequently in bi-monthly manner from new abstracts published at PubMed.

Specifications Table

**Table:** 

Subject	Health and medical sciences: Orthopaedics, Sports Medicine and Rehabilitation
Specific subject area	Orthopaedics, Total Joint Arthroplasty (TJA), Open Textual Dataset in TJA
Type of data	Raw, Analyzed, and Filtered
How the data were acquired	The scientific abstracts were collected from PubMed (https://pubmed.ncbi.nlm.nih.gov) using MeSH (Medical Subject Headings) terms.
Data format	Table (.xlsx and .csv format) JSON file
Description of data collection	To collect data from PubMed, we used MeSH terms within “MeSH Subheading” and “MeSH Major Topic”, and Publication Date from 01/01/2000 to 12/31/2022. The following MesH terms have been utilized to collect the data: arthroplasties, knee replacement; arthroplasties, replacement, knee; arthroplasty, knee replacement; arthroplasties, knee replacement; arthroplasties, hip replacement; arthroplasties, replacement, hip; arthroplasty, hip replacement; arthroplasties, hip replacement; arthroplasties, shoulder replacement; arthroplasties, replacement, shoulder; arthroplasty, shoulder replacement; arthroplasties, shoulder replacement; total joint arthroplasty; total joint arthroplasties; total knee arthroplasties; total knee arthroplasty; total hip arthroplasties; total hip arthroplasty; total shoulder arthroplasties; total shoulder arthroplasty.
Data source location	University of Pittsburgh Pittsburgh, PA 15260 USA
Data accessibility	Repository name: HexAI-TJAtxt Data identification number: 10.5281/zenodo.8124922 Direct URL to data: https://zenodo.org/records/8124922

## 1 Value of the Data

The “HexAI-TJAtxt” dataset is a valuable resource that not only consolidates existing scientific knowledge in TJA but also facilitates new discoveries and insights through computational analysis. The value of the HexAI-TJAtxt dataset could be listed as:

• **Research Advancement:** The HexAI-TJAtxt dataset provides a comprehensive collection of scientific abstracts related to total joint arthroplasty, assisting researchers, clinicians, health informaticians, and physicians to explore the upmost body of knowledge in the field and identify research gaps and areas. Individual scientists from different disciplines will delve into this dataset, gaining new insights and enhance their understanding of joint diseases, ultimately contributing to improved patient care and clinical outcomes in TJA.
• **Extensive Coverage:** The current textual dataset comprises 61,936 scientific abstracts from PubMed, providing a comprehensive collection of research on total joint arthroplasty (TJA) from the year 2000 to 2022, with bi-monthly updates from new abstracts that will be published at PubMed.
• **Supporting Evidence-Based Medicine:** The HexAI-TJAtxt empowers researchers and clinicians to make evidence-based decisions, facilitating literature reviews, meta-analyses, and systematic reviews related to TJA.
• **Interdisciplinary Research:** The HexAI-TJAtxt dataset encourages collaboration and knowledge exchange between researchers from different disciplines. Orthopedic surgeons, geneticists, epidemiologists, data scientists, AI scientists, and other experts can explore the dataset together, fostering interdisciplinary research and facilitating a holistic understanding of TJA.
• **Rapid Text Analytics:** The HexAI-TJAtxt dataset offers an opportunity for computational text analytics on a large-scale scientific literature. Researchers can employ natural language processing (NLP) techniques, machine learning algorithms, and other computational tools to extract valuable insights, discover patterns, and identify novel associations within the dataset, in a timely fashion.
• **Future Dataset Expansion:** The dataset will serve as a foundational data source for future dataset expansions, allowing for the inclusion of additional articles and updates to ensure the dataset remains up-to-date and representative of the research landscape in total joint arthroplasty.

## 2 Objective

With TJA being a prevalent and rapidly growing surgical procedure [1], the current dataset mainly aims to address the rising demand for timely and accessible comprehensive information derived from scientific literature. By assembling a large-scale collection of scientific abstracts from PubMed using MeSH terms, the HexAI-TJAtxt dataset not only provides a broad coverage of TJA-related agendas, but also enables computational text analytics to unlock new knowledge and insights in joint diseases. Furthermore, the dataset offers the potential for trend analysis and evaluation of changes in TJA clinical practices over time. By achieving these objectives, the HexAI-TJAtxt dataset contributes to significantly to the broader goals of better understanding TJA, improving patient care, and enhancing clinical and patient outcomes in this critical area of healthcare.

## 3 Data Description

**Fig. 1:**
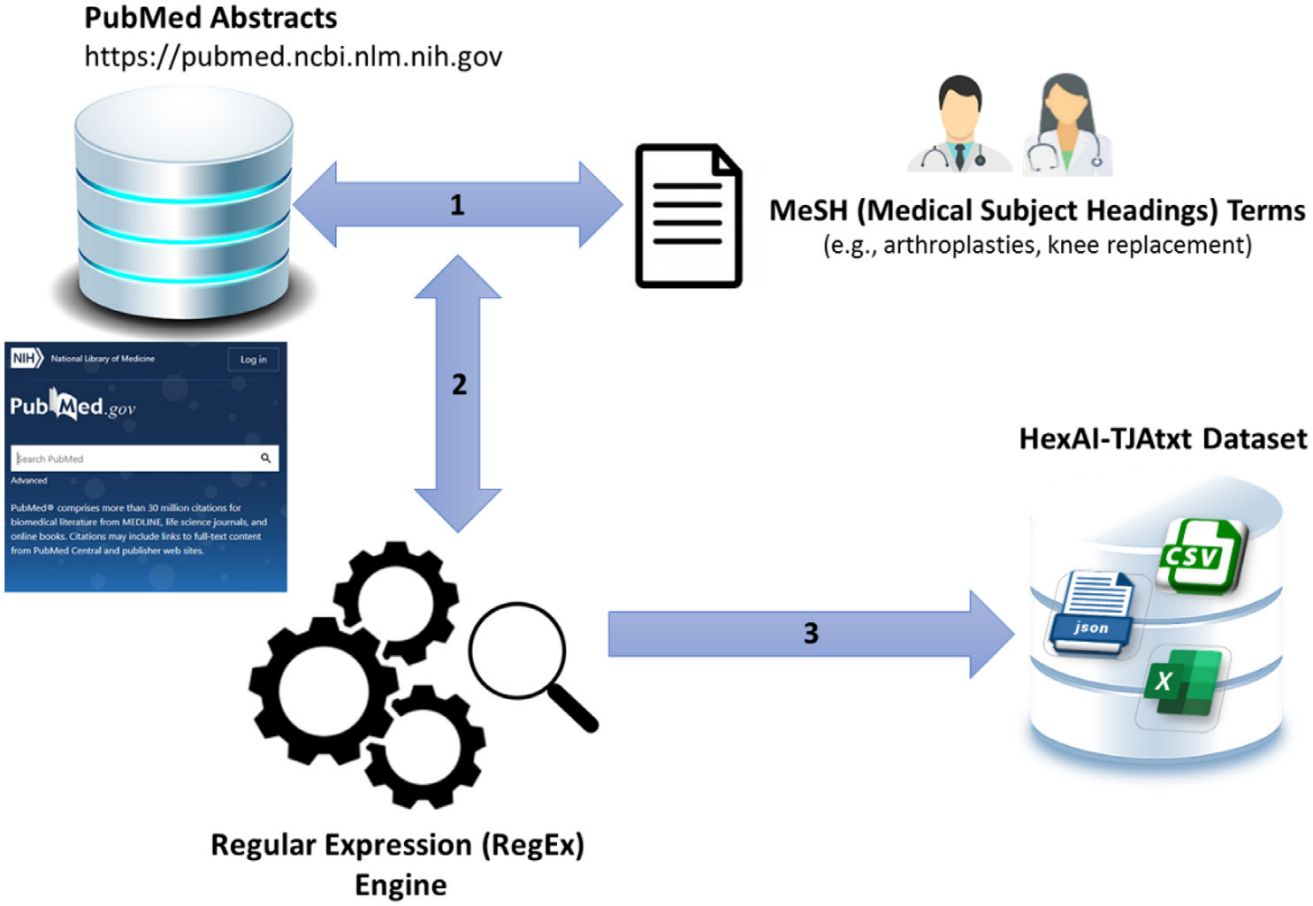
The proposed pipeline to build the HexAI-TJAtxt textual dataset. Utilizing this proposed pipeline, the HexAI-TJAtxt dataset will be frequently updated in a bi-monthly manner employing new abstracts published at PubMed.

Fig. 1 illustrates the proposed computational pipeline involved in constructing the HexAI-TJAtxt textual dataset. We have made a multidisciplinary team including physicians, computer scientists, and health informaticians to assemble the HexAI-TJAtxt textual dataset. Physicians in our team provided us with a list of relevant MeSH terms in the context of TJA, such as *arthroplasties, knee replacement; arthroplasties, replacement, knee;* as described in the specification table above. To collect data from PubMed, we then used the MeSH terms within “MeSH Subheading” and “MeSH Major Topic” combined with Publication Date from 01/01/2000 to 12/31/2022, using Advanced search available at PubMed focusing on Abstracts (text) only. Since scientific abstracts at PubMed often come with some meta-data (e.g., Author information, Copyright information, Date, DOI), we defined a set of regular expressions [[2], [3]] to automatically eliminate those meta-data and build a textual dataset that only covers abstracts body. For example, a regular expression of Comment (in|on) .* (Jan|Feb|Mar|Apr|May|Jun|Jul|Aug|Sep|Oct|Nov|Dec).{15,25}\. has been used to first find and then eliminate occurrences of comments that contain a specific date format. This regular expression expects a line that starts with “Comment in” or “Comment on” followed by any characters, then a month abbreviation (e.g., Jan, Feb, Mar, etc.), followed by 15 to 25 additional characters, and finally ending with a period. In doing so, we used Python programing, and all Python implementations are available at the GitHub repository. Finally, the textual dataset turned into three different structured data formats in Excel (xlsx, csv) and JSON, including:

(1) **HexAI-TJAtxt_June2023_XLSX.xlsx:** This Excel sheet comprised of three columns, including Year (Publication year), Abstract_ID (an identifier we've assigned to each abstract individually), and Abstract (Abstract body).
(2) **HexAI-TJAtxt_June2023_CSV.csv:** This file includes the same data as the above Excel sheet, however in .csv format.
(3) **HexAI-TJAtxt_June2023_JSON.txt:** This file includes the same data as the above Excel sheet, however in JSON format.

The HexAI-TJAtxt dataset comes with a limitation. There are some empty lines in the Excel/JSON files where the pipeline and the regular expressions got stuck finding the predefined patterns.

## 4 Experimental Design, Materials, and Methods

This section not only implements experimental validation around the quality and quantity attributes of the current textual dataset, but also introduces a list of applications of such a dataset. As the first experiment, we randomly selected 4000 abstracts from the current dataset, and trained a word embedding model as described in Task #1. Furthermore, we randomly selected several subsets of the dataset, and came with the set of abstracts shown in Appendix I, and then investigated two computational mechanisms to analyze it, one with the use of ChatGPT as a large language model (LLM), and the other with generating word clouds using those abstracts presented in Appendix I.

(1) Task #1: Word Embedding

**Fig. 2:**
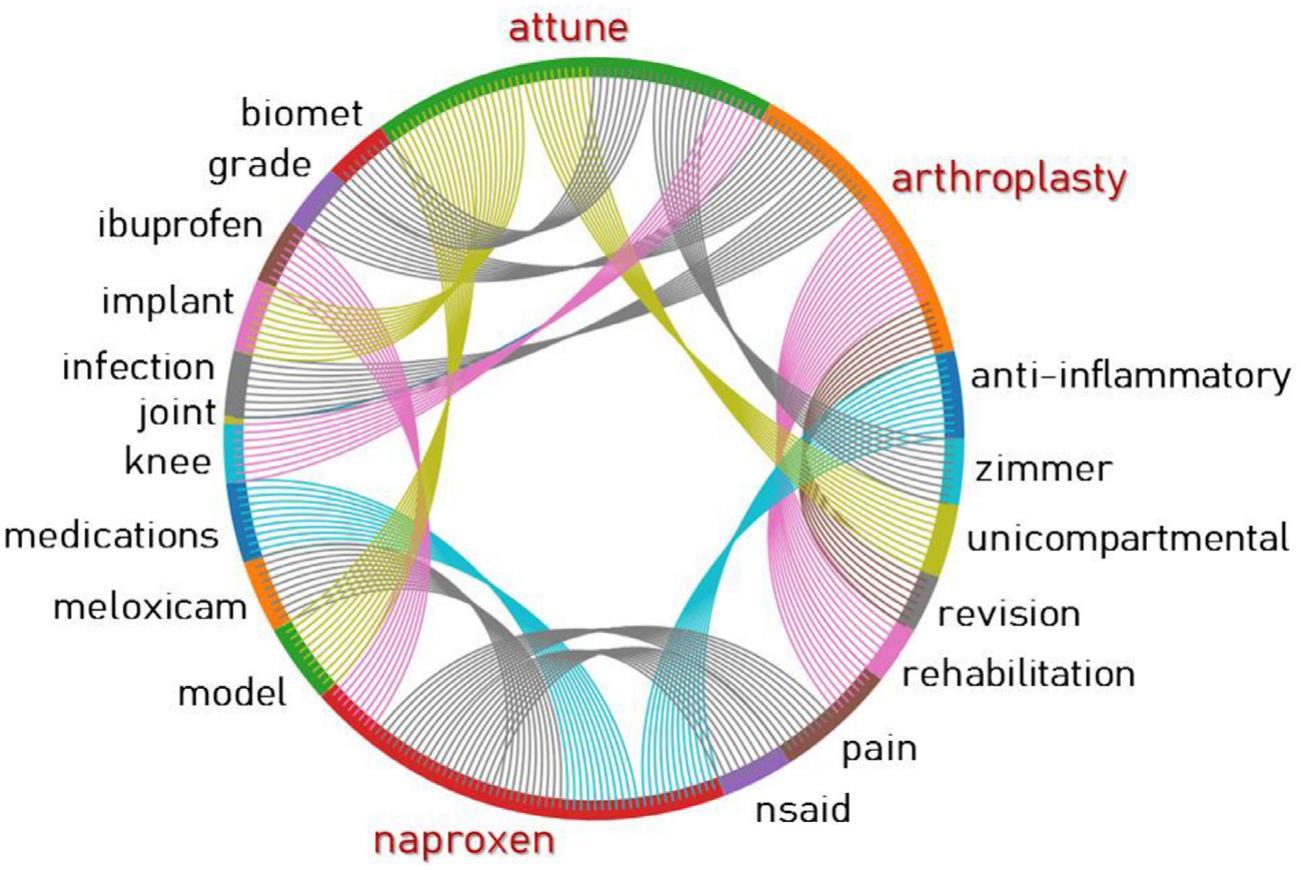
These scientific visualization results were obtained by searching three different terms, including “arthroplasty” as example of medical procedure, “naproxen” as an example of drug name, and “attune” as an example of implant brand/name. One can see, searching the term “arthroplasty’’ resulted in clinical meaningful and keywords, such as “join”, “pain”, “infection”, “grade”, “rehabilitation”, and “revision”. Furthermore, it also resulted in terms such as “anti-inflammatory”, “medication”, “meloxicam”, “nsaid”, “pain” and “ibuprofen” when we search for word similarities of “naproxen”.

In advanced natural language processing (NLP), the word embedding methods (e.g., Word2Vec) refer to a set of computational text analytics that map every individual word in a given corpus to a numerical vector [[4], [5], [6]]. For instance, applying word2vec word embedding method on a given medical text corpus (e.g., clinical notes, radiology reports), represents the word “femur” as a vector of [0.71, 0.52, -1.39, 1.12, 0.24], where such vector representation fits well with artificial neural nets (ANNs). Within this analysis, we applied Word2Vec algorithms [[4], [5]] on a random subset of the HexAI-TJAtxt dataset to capture word similarities, calculating the cosine similarity between word vectors. Generally speaking, word similarity measurement using the Word2Vec algorithm involves comparing the vector representations of words to determine their semantic similarity. We first trained a Word2Vec model using a random subset of the dataset, then obtained words vectors and computed similarity metric employing cosine similarity. Finally, we compared similarity scores and collected higher similarity scores where it indicates similar semantic meanings. As an example, Fig. 2 provides a scientific visualization result form an experiment in which we searched word dependencies and similarities for three different terms in the TJA setting, including “arthroplasty” as a medical procedure, “naproxen” as a drug name, and “attune” as an implant brand/name.

(2) Task #2: ChatGPT

Of late, powerful LLMs, such as GPT-2, GPT-3, GPT-4, PaLM2, LlaMA, and Bard have demonstrated impressive performance in various domains, such as healthcare, offering the potential to enhance healthcare accessibility, promote informed decision-making, and contribute to improved patient outcomes [[7], [8], [9], [10]]. This experiment incorporated the utilization of ChatGPT-3.5 developed by OpenAI (https://openai.com/, [11]), to solicit additional insights and perspectives based on asking the five following questions from ChatGPT to explore from the PubMed abstracts that was provided to it. The abstracts used for this experiment can be found in the Appendix I. Below, is the communication with Chat GPT-3.5 within each question performed on June 13, 2023.

Question #1: Extract drug names from the following text (Appendix I)

**Response generated by ChatGPT-3.5:**

**Fig. 3:**
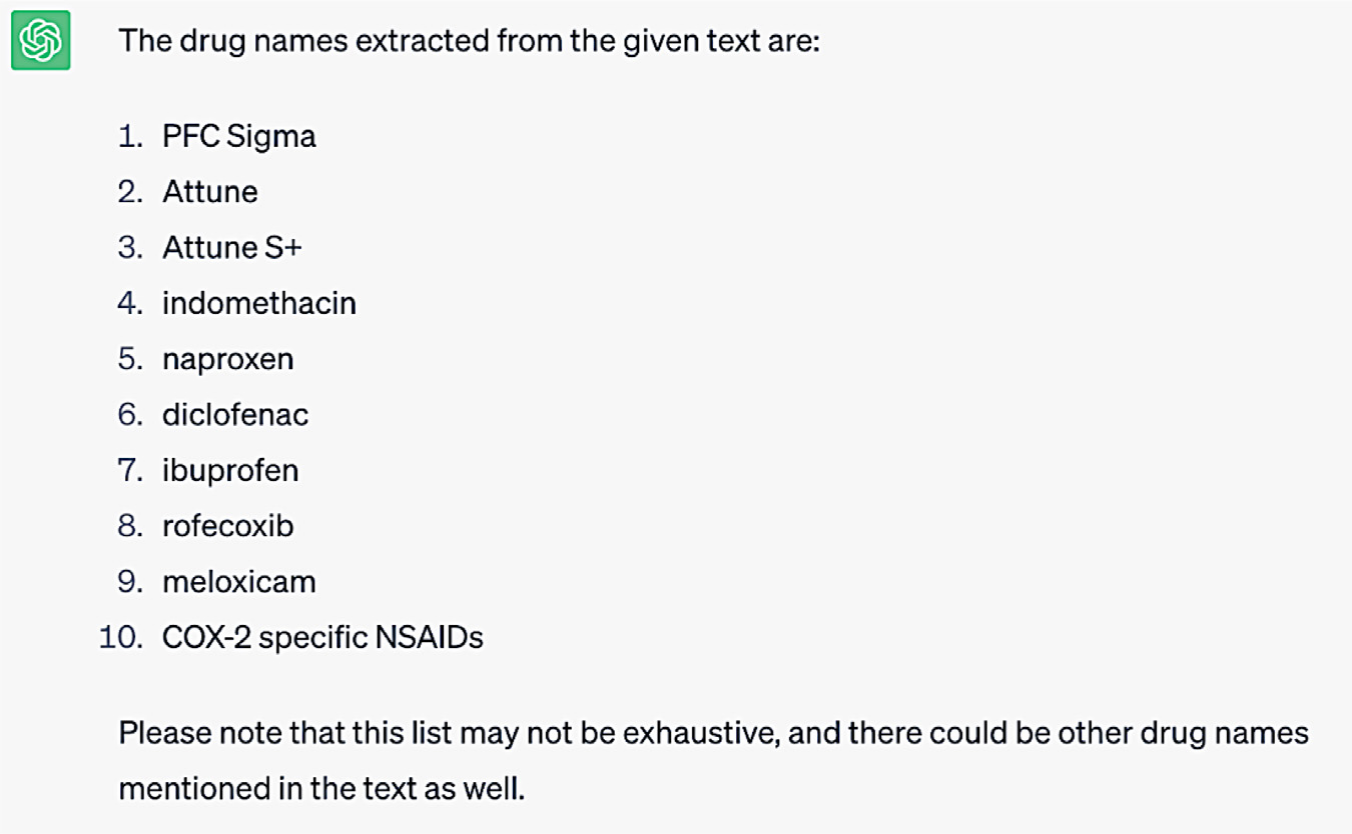
This is the response generated automatically by ChatGPT-3.5, when we sent a query of “extract drug names from the following text” using the text data available in Appendix I.

**Fig. 4:**
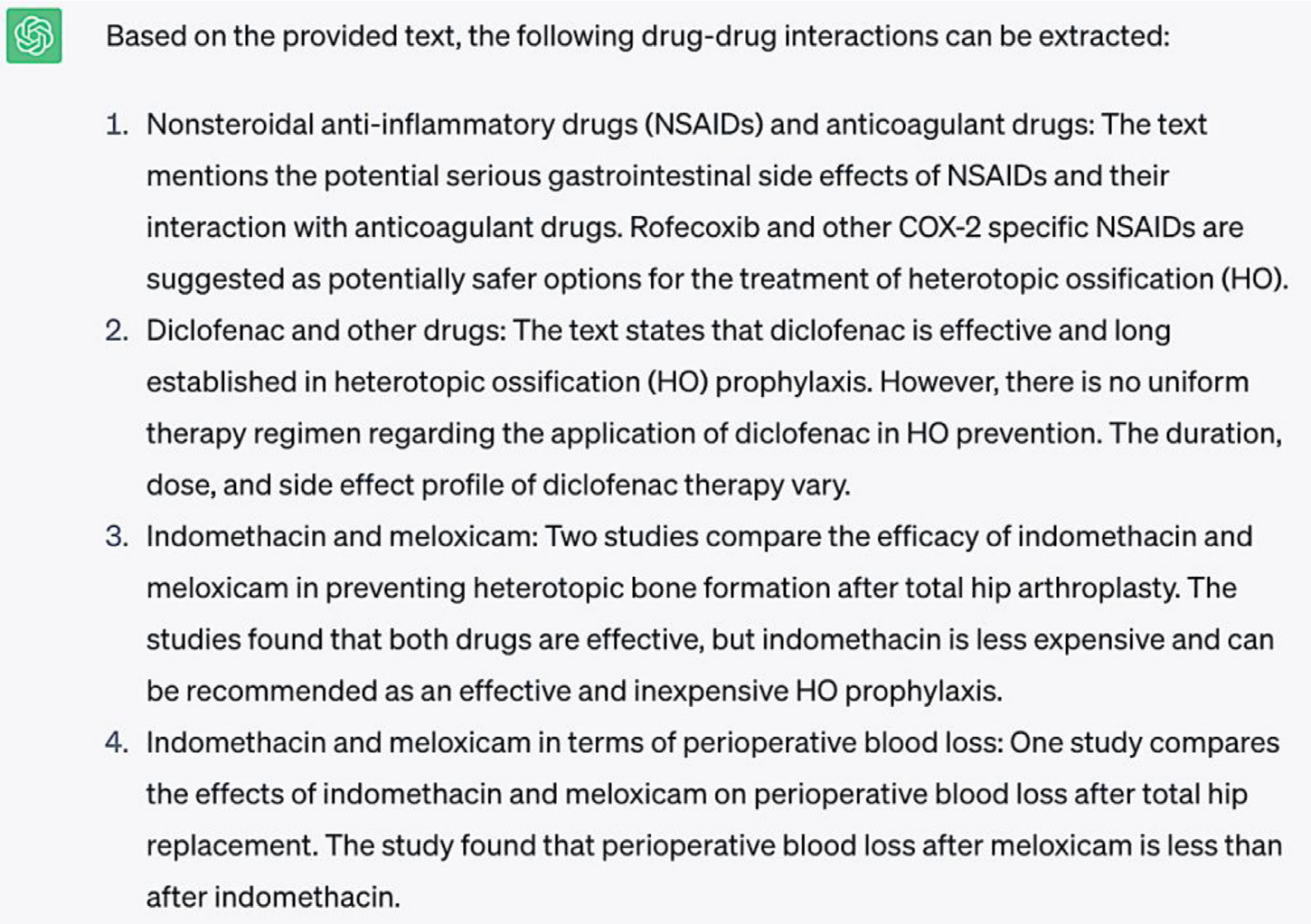
This is the response generated automatically by ChatGPT-3.5, when we sent a query of “extract drug-drug interactions from the following text” using the text data available in Appendix I.

**Fig. 5:**
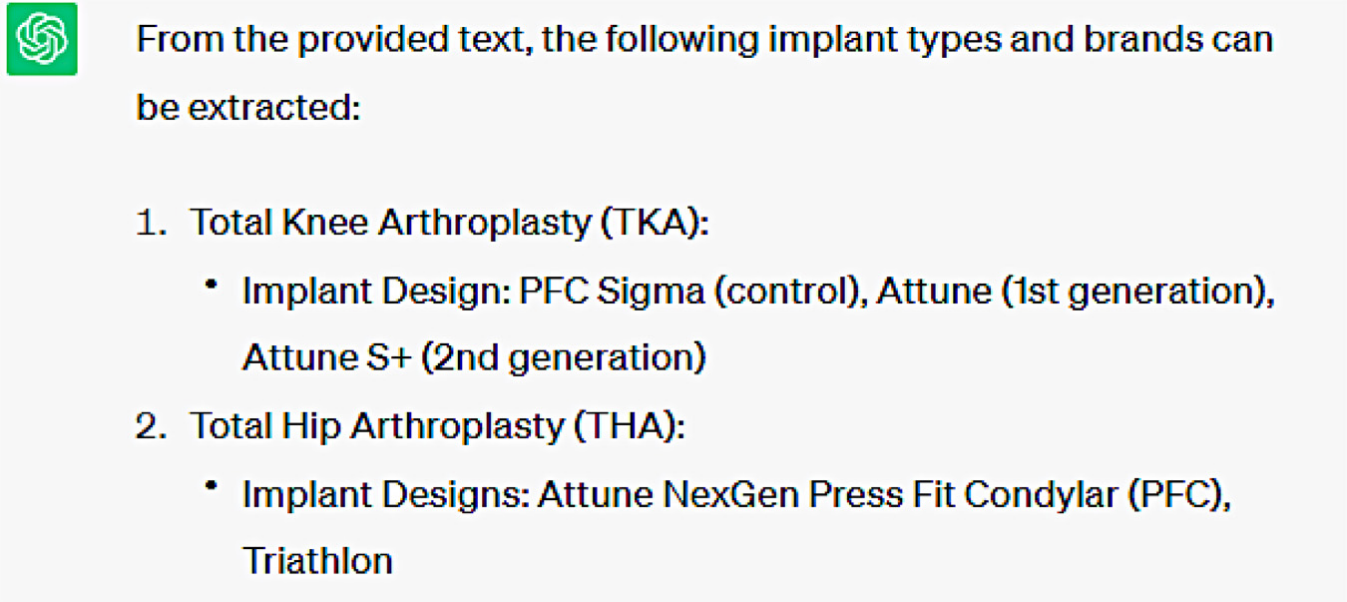
This is the response generated automatically by ChatGPT-3.5, when we sent a query of “extract implant types and brands from the following text” using the text data available in Appendix I.

**Fig. 6:**
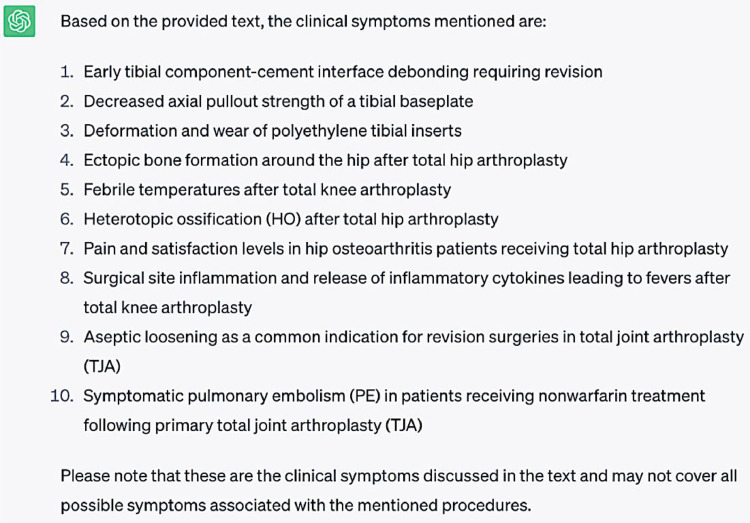
This is the response generated automatically by ChatGPT-3.5, when we sent a query of “extract clinical symptoms from the following text” using the text data available in Appendix I.

**Fig. 7:**
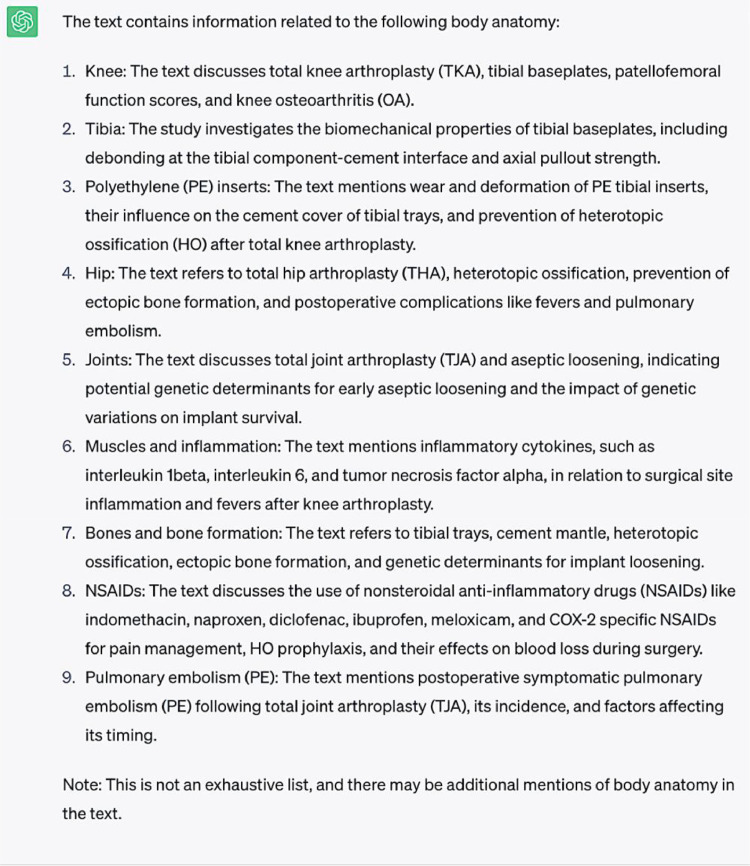
This is the response generated automatically by ChatGPT-3.5, when we sent a query of “body anatomy from the following text” using the text data available in Appendix I.

Within this query for extracting drug names, an overlap between drug names and implant brand names utilized in total joint arthroplasty was observed. For example, the first three entities extracted as drug names are in fact the names of total knee arthroplasty (TKA) implants. These issues with accurate drug name identification and implant name misidentification likely occurred for several reasons. For one, the language context in which implant brand names and drug names are utilized within these abstracts likely have some shared characteristics, as both may be described in the context of ability to provide pain relief or improve patient function. Furthermore, a valuable means of identifying drug names in the context of language includes references to the drug's physiological effects and pharmaceutical mechanism of action. Such characteristics are not associated with descriptions of orthopedic arthroplasty implants. However, within the body of orthopedic research literature, abstracts are less likely to discuss drug physiology or pharmacokinetics which may help distinguish these names from implant brand names. Furthermore, medications and implants are both interventions, broadly speaking, so it is possible ChatGPT-3.5 misidentified these entities due to an inability to make the relevant semantic distinction in this textual context. The communications between us and ChatGPT-3.5 are illustrated in Fig. 3, Fig. 4, Fig. 5, Fig. 6, Fig. 7.

**Question #2:** Extract drug-drug interactions from the following text (Appendix I)

**Response generated by ChatGPT-3.5:**

When queried to identify and abstract drug-drug interactions from the body of abstracts which were compiled, ChatGPT-3.5 performed relatively well. However, as is evident in the demonstrated response, the documented descriptions of drug-drug interactions more commonly discussed differences between drugs in treating a particular pathology. For example, the 3rd and 4th responses listed compared indomethacin and meloxicam in terms of their efficacy in preventing heterotopic ossification or on volume of perioperative blood loss, respectively. Only in the first response, where risk of side effects when drugs were combined was discussed, was the intent of the query accurately addressed. In essence, the capabilities of ChatGPT-3.5 at this time appear effective and promising in identifying drugs which may exert similar side effects, but the ability of reporting true drug-drug interactions which combine to increase risk of an adverse effect requires further optimization. Response 2 mentions “Diclofenac and other drugs”, but the explanation does not actually include interactions and instead mentions the effectiveness of diclofenac in prophylactically treating heterotopic ossification. The syntactic structure across all responses consistently lists “drug(s) A – drug(s) B”, so ChatGPT-3.5 seems to have appropriately responded to the initial part of the prompt. However, it is clear from these heterogeneous responses that ChatGPT-3.5 did not properly parse the “interactions” part of the question.

**Question #3:** Extract implant types and brands from the following text (Appendix I)

**Response generated by ChatGPT-3.5:**

In general, ChatGPT-3.5 proved effective at identifying and extracting arthroplasty implant brands. However, as demonstrated, the extraction process was not able to distinguish between total knee arthroplasty (TKA) and total hip arthroplasty (THA) implants. In particular, all of the THA implants listed were actually established TKA implant brands. This was likely a byproduct of the sample text included in the query including a larger predominance of abstracts discussing TKA implants. This was a limitation of not being able to provide a large body of diverse abstracts due to ChatGPT-3.5 processing limits. In the future, selection of a balanced set of abstracts in terms of discussion of TKA and THA implants will be implemented to evaluate how well this processor can distinguish the two different implants.

**Question #4:** Extract clinical symptoms from the following text (Appendix I)

**Response generated by ChatGPT-3.5:**

Overall, ChatGPT-3.5 was able to identify all clinical symptoms mentioned in the provided text. However, it was unable to completely distinguish between a symptom- a perceived manifestation of a condition by a patient- and an adverse event or sequela complication that may or may not lead to the patient experiencing symptoms. For example, while debonding, decreased pull out strength and heterotopic ossification are important sequalae to be aware of following TKA, they may not necessarily lead to symptoms experienced by the patient. This is perhaps a limitation of not expressly defining what a symptom is before asking ChatGPT-3.5 to identify the symptoms within the text. Additionally, some responses are more diagnostic findings than clinical symptoms. For example, response 3 describing the wear of part of a device. The question prompt is admittedly broad, so perhaps more relevant and accurate responses could have been generated with a question that specifically asked for clinical symptoms after total joint arthroplasty. In the future, a more accurate result could likely be thus obtained by asking a more specific query. This may include asking ChatGPT-3.5 what symptoms a patient may experience preoperatively that may serve as an indication for total joint arthroplasty.

**Question #5:** Extract body anatomy from the following text (Appendix I)

**Response generated by ChatGPT-3.5:**

ChatGPT-3.5 demonstrated reasonable success in distinguishing body anatomic regions, although it misidentified polyethylene inserts, NSAIDs, and pulmonary embolism in the provided list. In general, this suggests that although anatomic regions can be generally distinguished, the context provided by which ChatGPT-3.5 identifies anatomy overlaps with other terms such as total joint implants, medications, and complications. The inclusion of “NSAIDs” and “Pulmonary embolism” in this list demonstrate ChatGPT-3.5’s inability to make the proper semantic distinctions in this case. It is possible ChatGPT-3.5 interpreted the relation between NSAIDs and musculoskeletal pain management as being related to anatomy in some way and perhaps conflated pulmonary embolisms with anatomical structures of the pulmonary system. Certainly, this is potentially confounded using anatomic descriptors in certain terms, such as polyethylene inserts being described as tibial inserts, as well as pulmonary embolism including reference to the pulmonary system. Similarly, to this language processor's ability to distinguish the names of joint implants from medications, further developing a means to exclude terms with contrasting elements is required for better precision for these functions.

(3) Task #3: Word Clouds

**Fig. 8:**
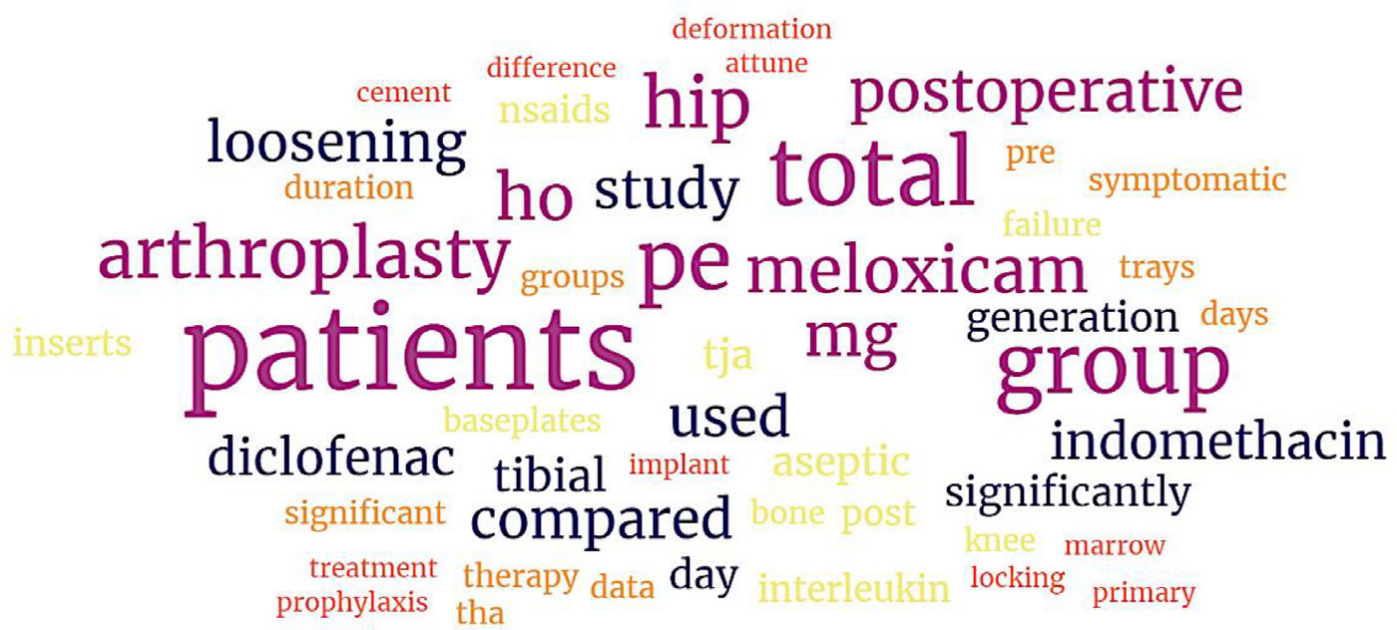
The word clouds generated using the text data available in Appendix I.

Within our last experiment, we are generating word clouds using those abstracts presented in Appendix I, provide a valuable overview of that text data. Word clouds are visual representations of textual information where the size of each word corresponds to its importance or frequency within that text. It assists identifying important keywords, understanding the main subjects, concepts, or trends within the body of text. Figure 8 presents the word clouds generated using the text data available in Appendix I, while removing all stop words (e.g., the, is, an), numbers, and special characters (e.g., $, #) was involved in the current word clouds visualization.

One can see in Fig. 8, the “patients” cloud indicates a significant focus from those abstracts (Appendix I) on patients, while “hip”, “arthroplasty”, “total” as perhaps total knee or total hip were also common terms being discussed in those studies. The current word clouds also illustrate “meloxicam”, “indomethacin”, and “diclofenac” as frequently specific NSAIDs mentioned in the text. In summary, the present word clouds provide a snapshot of the main topics and themes discussed in those abstracts shown in Appendix I, highlighting key terms related to surgical procedures, medications, treatment options, and the main research areas.

### 4.1 Limitations

The dataset is constructed from scientific articles available on PubMed, and its content is thus limited inherently to what is indexed on the PubMed. This may introduce a selection bias as it does not capture research findings published in other data sources. Additionally, it is important for users to be aware that the current dataset represents only a portion of the available research on Total Joint Arthroplasty (TJA). Moreover, the quality and the value of the abstracts and their content is reliant on the original articles from which they are sourced. Furthermore, the dataset consists of abstracts rather than full-text articles. While abstracts provide a summary of the research, they lack the depth and details that may be found in the full texts. Researchers may need to access the original articles for a more comprehensive understanding of the research. Finally, the HexAI-TJAtxt dataset relies on Medical Subject Headings (MeSH) terms for indexing and selection of relevant articles. The assignment of MeSH terms can be subjective and may not always fully capture the content of the articles. This subjectivity may impact the comprehensiveness of the dataset.

## Ethics Statements

N/A.

## CRediT author statement

**Soheyla Amirian:** Conceptualization, Study design, Methodology and code development, Data curation, Experimental validation, Original manuscript preparation, Reviewing and editing, Supervision. **Husam Ghazaleh:** Methodology and code development, regular expression design and implementation, Reviewing and editing, Scientific visualization. **Matthew Gong:** MeSH terms identification, Clinical validation, Original manuscript preparation, reviewing and editing. **Logan Finger:** MeSH terms identification, Clinical validation, Original manuscript preparation, Reviewing and editing. **Luke Carlson:** MeSH terms identification, Clinical validation, Original manuscript preparation, Reviewing and editing. **Johannes F. Plate:** Conceptualization, Study design, Original manuscript preparation, Supervision. **Ahmad P. Tafti:** Conceptualization, Study design, Methodology and code development, Data curation, Experimental validation, Original manuscript preparation, Reviewing and editing, Establishing and maintaining GitHub repository, Securing financial support, Supervision.

All authors read and approved the final manuscript.

## Data Availability

HexAI-TJAtxt (Original data) (Zenodo).

## Appendix I

**ChatGPT-3.5 Prompts:**

1. Extract drug names from the following text
2. Extract drug-drug interactions from the following text
3. Extract implant types and brands from the following text
4. Extract clinical symptoms from the following text
5. Extract body anatomy from the following text

Early clinical results of a new total knee arthroplasty (TKA) implant design show promise for improved outcomes and patellofemoral function scores. However, reports of early tibial component-cement interface debonding requiring revision have been published. This study investigated the biomechanical properties of three different tibial baseplates to understand potential causes of failure. PFC Sigma (control) Attune (1st generation) and Attune S+ (2nd generation) tibial baseplates were implanted into 4th generation sawbone tibia models using a standardized technique. Three of each baseplate were cemented with and without additional bovine bone marrow fat. All models were tested to failure with measured axial distraction force. Implant type presence or absence of bovine marrow and load to failure were all recorded and compared. Two-way ANOVA followed by post-hoc pairwise comparisons were used to determine statistical significance which was set to P < .05. The 2nd generation tibial baseplates required significantly more force to failure. The presence of bovine marrow significantly reduced the pullout force of the implant designs overall. No significant difference was detected between the 1st generation and control baseplates. Failure mode for each model was also noted to be different irrespective of the presence or absence of bone marrow fat. The 2nd generation baseplates required significantly more force to failure compared with older designs. The presence of bone marrow during cementation of a tibial base plate significantly decreased axial pullout strength of a tibial baseplate in this laboratory model. All 1st generation baseplates exhibited debonding at the cement-implant interface.

The aim of this study was to investigate whether wear and backside deformation of polyethylene (PE) tibial inserts may influence the cement cover of tibial trays of explanted total knee arthroplasties (TKAs). At our retrieval center we measured changes in the wear and deformation of PE inserts using coordinate measuring machines and light microscopy. The amount of cement cover on the backside of tibial trays was quantified as a percentage of the total surface. The study involved data from the explanted fixed-bearing components of four widely used contemporary designs of TKA (Attune NexGen Press Fit Condylar (PFC) and Triathlon) revised for any indication and we compared them with components that used previous generations of PE. Regression modelling was used to identify variables related to the amount of cement cover on the retrieved trays. A total of 114 explanted fixed-bearing TKAs were examined. This included 76 used with contemporary PE inserts which were compared with 15 used with older generation PEs. The Attune and NexGen (central locking) trays were found to have significantly less cement cover than Triathlon and PFC trays (peripheral locking group) (p = 0.001). The median planicity values of the PE inserts used with central locking trays were significantly greater than of those with peripheral locking inserts (205 vs 85 microns; p < 0.001). Attune and NexGen inserts had a characteristic pattern of backside deformation with the outer edges of the PE deviating inferiorly leaving the PE margins as the primary areas of articulation. Explanted TKAs with central locking mechanisms were significantly more likely to debond from the cement mantle. The PE inserts of these designs showed characteristic patterns of deformation which appeared to relate to the manufacturing process and may be exacerbated *in vivo*. This pattern of deformation was associated with PE wear occurring at the outer edges of the articulation potentially increasing the frictional torque generated at this interface.

Non steroidal anti-inflammatory drugs (NSAIDs) and prophylactic radiotherapy can prevent ectopic bone formation around the hip after total hip arthroplasty. We retrieved from Medline Embase and the Cochrane Register (clinical) trials and other relevant literature on the prevention of heterotopic ossification (HO) from 1990-2002 for further review. Review of these clinical trials shows that HO is effectively prevented by a postoperative NSAID treatment with indomethacin for at least seven days. The best evidence is available for indomethacin although naproxen diclofenac and ibuprofen are also well documented. Short term use of ibuprofen is not effective. If NSAIDs are contraindicated preoperative or postoperative radiotherapy is a very effective therapeutic option to prevent HO. Because of the potential of serious gastrointestinal side effects of NSAIDs and their interaction with anticoagulant drugs rofecoxib and other COX-2 specific NSAIDs may be a safer option for the treatment of HO. However randomized controlled studies are needed to confirm the results of the rofecoxib study.

Diclofenac is 1 of the most used drugs in HO prophylaxis because it is effective and long established. However, there is still no uniform therapy regimen in terms of duration dose and side effect profile regarding the application of diclofenac in HO prevention. We have therefore conducted the first systematic review investigating diclofenac for HO prophylaxis after hip arthroplasty. The aim of this study is to assess the efficacy dose and duration of diclofenac therapy in preventing HO after total hip arthroplasty (THA). According to the PRISMA Guidelines we performed a systematic literature search in EMBASE via Ovid in MEDLINE via PubMed and in the Cochrane Library addressing all studies in English and German regarding the prophylaxis of HO with diclofenac after THA. We identified 731 potential studies and included 6 randomized controlled trials with 957 patients. The studies were heterogeneous with regard to duration of therapy dose comparative group and follow-up period. The therapy duration ranged from 9 to 42 days the applied diclofenac doses ranged from 75 mg to 150 mg daily. Patients treated with diclofenac showed a significant reduction in the total incidence of HO regarding to the Brooker Classification compared to placebo and no clinically relevant ossifications occurred (Brooker III and IV). Diclofenac is efficacious in the prevention of HO and can be used routinely after THA. The existing data indicates that a minimum dose of 75 mg diclofenac per day started on the first postoperative day for a minimum of 9 days is needed to prevent HO with an acceptable incidence of side effects such as gastrointestinal symptoms.

This study aimed to compare the analgesic effect patients' satisfaction tolerance and hip-joint function recovery by preoperative meloxicam versus postoperative meloxicam in treating hip osteoarthritis (OA) patients receiving total hip arthroplasty (THA). 132 hip OA patients who underwent THA surgery were allocated into postoperative analgesia (POST) and preoperative analgesia (PRE) groups at a 1:1 ratio. In the PRE group patients took meloxicam 15 mg at 24 h pre-operation 7.5 mg at 4 h 24 h 48 h and 72 h post-operation; in the POST group patients received meloxicam 15 mg at 4 h post-operation then 7.5 mg at 24 h 48 h and 72 h post-operation. Furthermore, postoperative pain consumption of patient-controlled analgesia (PCA) overall satisfaction and adverse events were evaluated within 96 h post-operation; meanwhile Harris hip score was assessed within 6 months post-operation. Pain VAS at rest at 6 h 12 h 24 h and pain VAS at passive movement at 6 h 12 h were decreased in PRE group compared to POST group. In addition, additional consumption of PCA and the total consumption of PCA were both reduced in PRE group compared to POST group. Additionally overall satisfaction in PRE group was higher at 24 h 48 h and 72 h compared to POST group. While Harris hip score was of no difference between POST group and PRE group at M3 or M6. Besides no difference in adverse events incidence was found between the two groups. In conclusion preoperative meloxicam achieves better efficacy and similar tolerance compared to postoperative meloxicam in hip OA patients post THA.

Febrile temperatures commonly are seen after total knee arthroplasty, but their source and importance are unclear. The goal of the current study was to determine whether such fevers are part of the normal physiologic response to surgery mediated by inflammatory cytokines. In 20 patients who had total knee arthroplasty serum and wound drain fluid samples were collected preoperatively and at 1 6 24 and 48 hours postoperatively; oral temperatures were measured postoperatively every 4 h for 3 days. Concentrations of interleukin 1beta interleukin 6 and tumor necrosis factor alpha in the samples were measured via enzyme-linked immunosorbent assays and compared in patients who did and did not have fevers develop (>or=38.5 degrees C). Gender age operative time amount of blood loss or drain output anesthesia type drop in hematocrit and transfusion administration were not associated with fever. Significant increases were seen postoperatively in drain fluid concentrations of interleukin 1beta and interleukin 6 and in serum concentrations of interleukin 6. Patients who were febrile had significantly higher drain and serum interleukin 6 concentrations than patients who were afebrile. These findings suggest that fevers seen after total knee arthroplasty are at least partly the result of surgical site inflammation and subsequent local and systemic release of the endogenous pyrogen interleukin 6.

Probands underwent cementless total hip replacements at the Orthopaedic Department Hospital Wiener Neustadt from January 1997 to January 1998 did not take NSAID up to 4 weeks preoperatively and had no NSAID contraindications. Patients were separated into two groups by different hospitalization floors. All patients selected for this study suffered from primary or secondary coxarthrosis. Data were collected as a prospective randomized parallel group study. Patients were given 50 mg indomethacin 2 times daily (n=58) vs 7.5 mg meloxicam (n = 58) in a 12-day treatment course. A two-sided Cochran-Armitage trend test showed no statistically significant difference (p < 0.05) for one of the drugs regarding influence on ectopic bone formation according to the grading system of Brooker et al. Our study demonstrates that there is no statistically significant trend that indomethacin or meloxicam protects a hip arthroplasty better from heterotopic bone formation. We prefer indomethacin therapy because it is almost half the price of meloxicam therapy, and we recommend indomethacin in a 12-day treatment course given 50 mg 2 times daily as an effective inexpensive and easily administered HO prophylaxis.

Data from 200 patients who underwent total hip replacement were studied. Two NSAIDs were compared: indomethacin 50 mg (n = 82) and meloxicam 15 mg (n = 86). Both NSAIDs were given orally 1 h before surgery. The two groups were not different with respect to age gender ASA class or duration of surgery. When indomethacin was used preoperatively intraoperative blood loss was 623 +/- 243 mL (mean +/- SD) and postoperative blood loss 410 +/- 340 mL. After meloxicam these values were 524 +/- 304 mL and 358 +/- 272 mL respectively. Total perioperative blood loss after meloxicam was 17 % (P < 0.05) less than that observed after indomethacin. Perioperative blood loss after meloxicam is less than after indomethacin. These *in vivo* findings are consistent with *in vitro* results using selective COX 2 NSAIDs.

Total joint arthroplasty (TJA) is one of the most frequent surgical procedures performed in modern hospitals and aseptic loosening is the most common indication for revision surgeries. We conducted a systemic exploration of potential genetic determinants for early aseptic loosening. Data from 423 patients undergoing TJA were collected and analyzed. Three analytical groups were formed based on joint arthroplasty status. Group 1 were TJA patients without symptoms of aseptic loosening of at least 1 year group 2 were patients with primary TJA and group 3 were patients receiving revision surgery because of aseptic loosening. Genome-wide genotyping comparing genotype frequencies between patients with and without aseptic loosening (group 3 vs groups 1 and 2) was conducted. A case-control association analysis and linear modeling were applied to identify the impact of the identified genes on implant survival with time to the revision as an outcome measure. We identified 52 single-nucleotide polymorphisms (SNPs) with a genome-wide suggestive P value less than 10-5 to be associated with the implant loosening. The most remarkable odds ratios (OR) were found with the variations in the IFIT2/IFIT3 (OR 21.6) CERK (OR 12.6) and PAPPA (OR 14.0) genes. Variations in the genotypes of 4 SNPs-rs115871127 rs16823835 rs13275667 and rs2514486-predicted variability in the time to aseptic loosening. The time to aseptic loosening varied from 8 to 16 years depending on the genotype indicating a substantial effect of genetic variance. Development of the aseptic loosening is associated with several genetic variations, and we identified at least 4 SNPs with a significant effect on the time for loosening. These data could help to develop a personalized approach for TJA and loosening management.

The purpose of this study is to investigate the incidence and timing of postoperative symptomatic pulmonary embolism (PE) in patients receiving nonwarfarin treatment following primary total joint arthroplasty (TJA) to clarify the appropriate duration of postoperative VTE prophylaxis. We retrospectively reviewed the medical records of 11 148 patients who underwent primary TJA including total knee arthroplasty and total hip arthroplasty at our institution between January 2012 and March 2019. The median postoperative day of diagnosis of symptomatic PE and the interquartile range for day of diagnosis were determined. Multivariate Cox proportional hazards modeling was used to test the difference of timing for PE based on demographics and comorbidities.  The overall 90-day rate of symptomatic PE was 0.71 %. The median day of diagnosis for symptomatic PE was 3 days postoperatively (interquartile range 2–7 days). Factors showed statistical significance on multivariate analysis in association with earlier timing of PE occurrence in patients with atrial fibrillation diabetes mellitus coronary heart disease and history of stroke.  The vast majority of symptomatic PE occurs in the early postoperative period after TJA and atrial fibrillation diabetes mellitus coronary heart disease and history of stroke were independent factors affecting the timing of symptomatic PE.
